# Digital clubbing occurring in intrathoracic Hodgkin lymphoma: A case report

**DOI:** 10.1097/MD.0000000000018388

**Published:** 2019-12-20

**Authors:** Xiaoyan Gai, Wei Yang, Hong Zhu, Yongchang Sun

**Affiliations:** Department of Pulmonary and Critical Care Medicine, Peking University Third Hospital, Beijing, China.

**Keywords:** digital clubbing, intrathoracic lesion, lymphoma

## Abstract

**Rationale:**

Digital clubbing is rarely associated with lymphoma. This study reports a case of intrathoracic Hodgkin lymphoma with digital clubbing and reviews the current literature regarding the clinical characteristics of this condition.

**Patient concern:**

A 21-year-old woman presented with a 3-month history of cough and 1 month of fever, with apparent digital clubbing. A computed tomography (CT) scan of the lungs revealed a large pulmonary mass.

**Diagnosis:**

A CT-guided transthoracic needle biopsy was conducted. Pathology determined a diagnosis of Hodgkin lymphoma.

**Interventions:**

The patient underwent 6 courses of chemotherapy and intensification, followed by autografting.

**Outcomes:**

The patient recovered and a complete hematological remission was obtained. The patient is alive with no evidence of disease 60 months after diagnosis, with the digital clubbing of the fingers and toes completely resolved.

**Conclusion:**

Patients with digital clubbing and intrathoracic lesions need to be examined carefully to determine tumor malignancy.

## 1 Introduction

First described by Hippocrates in 400 B.C., digital clubbing is regarded as one of the most ancient clinical signs in medicine. It is characterized by an increase in nail plate convexity, with focal and bulbous enlargement of the distal phalange, and results in a pestle-like appearance of the fingers and toes. Digital clubbing is associated with a variety of diseases, including infections, inflammatory disease, cyanotic heart disease, and primary or metastatic pulmonary malignancies.^[1]^ Although lung cancer has been known to be associated with digital clubbing, it is rarely associated with lymphoma. Herein, we report a patient with digital clubbing and intrathoracic lymphoma. The patient was treated with systemic chemotherapy with complete remission and intensification followed by autografting, and a subsequent complete resolution of the digital clubbing.

## 2 Case presentation

**Figure 1:**
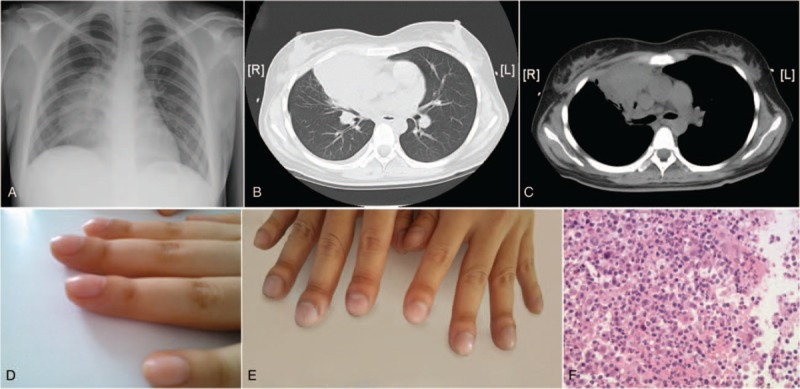
(A) Posteroanterior chest X-ray and (B, C) thoracic computed tomography imaging, which display a large lung mass. (D, E) Digital clubbing present in the hands of the patient. (F) Hematoxylin and eosin staining of the lung mass, showing nodular sclerosis Hodgkin lymphoma (magnification ×40).

A 21-year-old woman was admitted to our hospital with a 3-month history of productive cough, and a 1-month history of fever. The patient was first admitted to a local hospital, 2 months prior. Routine blood tests showed a white blood cell count of 19.4 × 10^9^/L and a neutrophil proportion of 85.5%. Chest X-ray and computed tomography (CT) of the chest showed a lung mass with right pleural effusion (Fig. 1 A, B, and C). The initial consideration was community-acquired pneumonia. The patient was administered intravenous anti-infective treatment for almost 1 month without improvement. The patient was then transferred to a tuberculosis specialty hospital. A purified protein derivative skin test result was negative. Bronchoscopy showed mucosal congestion; however, a cytologic examination was negative for tuberculosis. The patient was given intravenous antibiotics for another 15 days; however, the fever persisted. Subsequently, the patient underwent 3 weeks of quadruple anti-tuberculosis treatment, with the fever symptoms continuing to persist. A lung CT scan showed that the shadow mass had increased in size. Thus, the patient was admitted to our hospital. Her past history and family history were unremarkable.

Examination upon admission showed a temperature of 38°C, pulse of 110 beats/minute, respiration of 18 beats/minute, and blood pressure of 117/82 mm Hg. Systemic superficial lymph nodes showed no signs of enlargement. Breathing sounds were clear in both lungs. Breath sounds in the right lung seemed slightly lower, yet no dry or wet rales were heard from either lung. The heart rate was 110 beats/minute, with normal rhythm. There was no hepatosplenomegaly. Most notably, clubbing of the fingers and toes was noticed (Fig. 1D, and E). The admission diagnosis of the patient was: fever, lung mass shadow of unknown origin, lymphoma, or lung cancer.

Hematology assessment showed: white blood cell count of 24.18 × 10^9^/L with neutrophil proportion of 87%, hemoglobin level of 99 g/L, platelet count of 353 × 10^9^/L, and an erythrocyte sedimentation rate of 87 mm/h. No abnormalities were observed in liver function, renal function, cardiac enzyme level, blood glucose level, or blood lipid level. The concentration of β2-microglobulin was 1.4 mg/L. Multiple bacterial cultures yielded negative results. B-ultrasound did not detect any swelling of the superficial lymph nodes. Abdominal B-ultrasound scan of the liver and spleen did not detect any abnormalities. A positron emission tomography scan showed an isolated hypermetabolic uptake in the right lung with violations of the mediastinum. The SUVmax was 14.2. The likelihood of malignant lesions was defined as high. No other identifiable adenopathy was seen. Bone marrow aspiration and a biopsy was performed which indicated myeloid hyperplasia. Lymphoma or lung cancer was suspected to be the main cause of symptoms. Therefore, a CT-guided transthoracic needle biopsy was conducted. The pathology report supported nodular sclerosis Hodgkin lymphoma (Fig. 1F).

The patient was transferred to the Hematology Department for further treatment. A complete hematological remission was obtained after 6 courses of chemotherapy and intensification, followed by autografting. The digital clubbing resolved during the second course of chemotherapy. The patient is alive with no evidence of disease 60 months after diagnosis, with the digital clubbing of the fingers and toes completely resolved. Written informed consent was obtained from the patient for publication of the case.

## 3 Literature search and review

We performed a computer-assisted search of PubMed, Embase, and Wanfang databases from 1970 to July 2017 using the keywords “intrathoracic Hodgkin lymphoma and digital clubbing.” We also used other keywords including cancer, lymphoma, pathogenesis, clinical characteristics, treatment, and outcome. The case presentations, radiographic findings, diagnoses, management, and patient outcomes are described below.

Digital clubbing is rarely associated with lymphoma and is described in the literature only as case reports (2–11) (Table 1). Including our case, 14 patients were analyzed, with 6 females (42.9%) and 8 males (57.1%). The average patient age was 20.0 years (standard deviation, SD = ±9.3 years) and ranged from 8 to 42 years. The clinical presentations included cough (6 patients), fever (4), weight loss (4), swelling and pain of knees and ankles (4), dyspnea (3), neck mass (3), clubbing of the fingers and toes bilaterally with pain (2) or without pain (2), fatigue (1), night sweats (1), hemoptysis (1), and abdominal pain (1). Radiographic manifestations varied, including mediastinal mass (2 patients), lung mass with cavitation (1) or without cavitation (2), pulmonary consolidation with cavitation (1), mediastinal lymphadenopathy (7), and pleural effusion (2). Patients underwent biopsy, including lymph node biopsy (5), thoracotomy (3), CT-guided lung biopsy (2), mediastinoscopy (1), autopsy (2), and not available (1). All diagnoses were based on pathology, with pathologic types including nodular sclerosis (6), mixed cellularity (3), and lymphocyte depletion (1). In 4 cases, detailed pathologic types of Hodgkin were not available. Eight patients responded to chemotherapy and radiotherapy and 3 patients died. In the other 3 cases, outcomes were not available. Digital clubbing resolved in all 8 patients who responded to treatment.

**Table 1:**
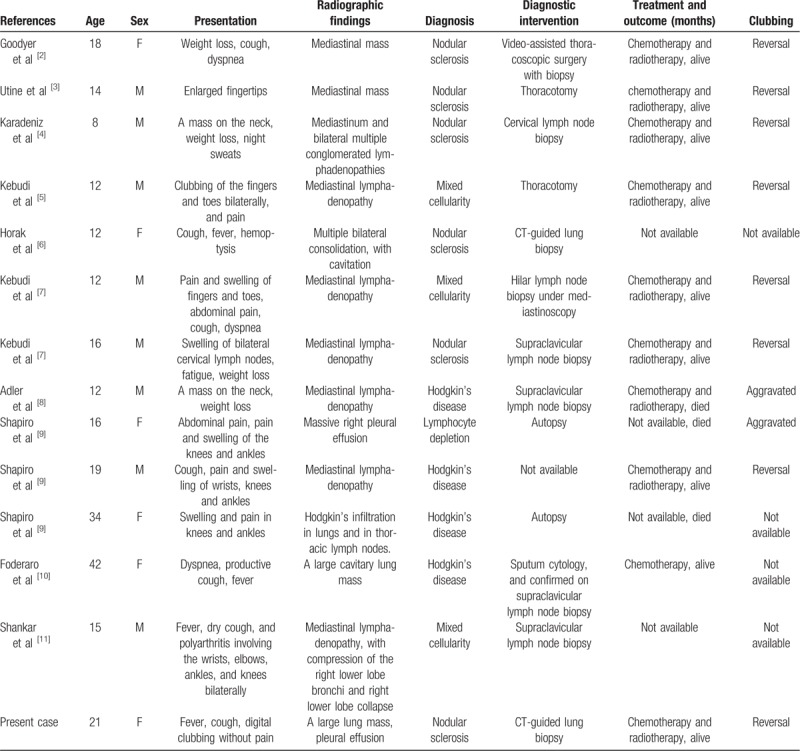
Summary of reported cases of Hodgkin lymphoma and digital clubbing since 1970.

## 4 Discussion and conclusion

We reported a 21-year-old woman with a productive cough for 3 months and fever for 1 month. Physical examination showed clubbing of all fingers and toes. CT imaging showed a mass in the lung region. The diagnosis was assumed to be lung cancer or lymphoma after admission. CT-guided biopsy was performed, and a subsequent diagnosis of nodular sclerosis Hodgkin lymphoma was determined. In this patient, the tumor was limited to the lung, who was diagnosed as primary pulmonary lymphoma. Primary pulmonary lymphoma is a rare disease, and its diagnosis is often delayed because of atypical clinical presentation and slow progression.^[12,13]^

The patient had significant signs of digital clubbing. If the clubbing was graded as a 5-step process, it was estimated as Grade 4.^[1]^ Digital clubbing is also known as Hippocratic finger. The symptoms are finger- or toe-tip hyperplasia and hypertrophy, which reflects the enlargement of the nail bed and distal phalanges due to increased blood flow and connective tissue, with subsequent remodeling of the bony tips of the digits.^[14,15]^ Digital clubbing can be an isolated finding or may occur as part of hypertrophic osteoarthropathy (HOA). HOA is a medical condition characterized by abnormal proliferation of the skin and periosteal tissues involving the extremities and is characterized by 3 clinical features: digital clubbing, periostosis of the tubular bones, and synovial effusion. Secondary HOA may also present as the full spectrum of HOA or as isolated finger clubbing.^[16]^ Secondary HOA has been associated with other neoplastic, pulmonary, cardiac, gastrointestinal, infectious, endocrine, and psychiatric conditions,^[16]^ and approximately 80% are found with primary or metastatic pulmonary malignancies, which explains why this condition was formerly referred to as hypertrophic pulmonary osteoarthropathy.^[14]^ In addition, patients with non-small cell carcinoma of the lung (NSCLC) have been reported to be more likely to have digital clubbing than small cell carcinoma of the lung (SCLC).^[17,18]^ Sridhar et al reported 111 patients with lung cancer; 32 of which demonstrated clubbing (29%), 35% of patients who had NSCLC and 4% of patients with SCLC had clubbing.^[17]^ Although many clinicians are familiar with the strong association between secondary HOA (including digital clubbing) and pulmonary neoplasms, there were occasional reports of lymphoma patients with digital clubbing, mostly as case reports.

We searched the literature for digital clubbing in Hodgkin lymphoma cases. In 2009, Goodyer et al reported a case of an 18-year-old woman with symptoms of cough and weight loss over a 2-year period.^[2]^ On examination, the patient had obvious bilateral clubbed fingers and toes. CT imaging showed a large mediastinal mass. A video-assisted thoracoscopic surgery lung biopsy helped to diagnose the patient with nodular sclerosis Hodgkin lymphoma. The patient was without joint pain, but with symptoms of tibia and fibula distal periosteal thickening, which was considered to be HOA. Utine et al reported a 14-year-old boy diagnosed with nodular sclerosis Hodgkin lymphoma.^[3]^ He also showed significant finger clubbing but without joint pain. This patient underwent a combination of radiotherapy and chemotherapy, with the lymphoma going into remission. Some patients had symptoms of joint pain, with joint effusion also possibly detected, often with new bone growth under the periosteum. Further determination was required to rule out inflammatory arthritis.^[14]^

The cause of digital clubbing has been a medical mystery for over 2400 years.^[1,19]^ Numerous hypotheses regarding the pathophysiology of digital clubbing have been proposed in the past decades. The most promising hypothesis has been that of Dickinson et al; they proposed the trigger mechanism could be local activation of platelet-endothelial cells causing an increased release of fibroblast growth factors (such as platelet-derived growth factor.^[1,20]^ The pulmonary megakaryocytes did not split into platelets, instead they circulated to the distal limbs and released growth factors. Tumor tissue produced a releasing factor (vascular endothelial growth factor) to stimulate the formation of digital clubbing and HOA.^[1,14,20]^ Isolated digital clubbing may precede the development of HOA.^[21]^ Some study showed elevated Prostaglandin E2 may also be linked to clubbing and HOA in lung cancer patients.^[22]^ Although the pathogenesis of clubbing in lymphoma is unknown, proposed mediators such as estrogens, circulatory and neurogenic factors, growth hormones produced by tumors, and “toxic substances or hormones,” which were inadequately cleared from the circulation due to pulmonary arteriovenous shunts, have been postulated to play a role by stimulating periosteal growth.^[1,3,4,23]^

Clubbing has been studied widely as a prognostic factor for its underlying malignancy,^[24]^ In 2017, Ciment et al reported a 59-year-old woman with lung cancer with digital clubbing that developed over a 1-year period, and the digital clubbing diminished after an impressive response to cancer treatment.^[25]^ This is another feature of digital clubbing, and it is often reversible after successful treatment of the underlying malignancy, with most patients with concurrent internal malignancy and digital clubbing having either died or showed extensive metastases.^[1–4]^

Based on the previous findings, hypertrophic pulmonary osteoarthropathy was more common in adults and rarely in teenagers. Lung cancer could induce endocrine changes and cause abnormalities in the joints, and this may also occur in Hodgkin lymphoma. This case has deepened the understanding of Hodgkin lymphoma symptoms, in that it could be combined with finger clubbing. The appearance of an intrathoracic mass, together with digital clubbing, should be treated with vigilance as a potential case of lymphoma.

In conclusion, although coexistence of digital clubbing with lymphoma is rarely reported, it is important to consider the possibility of lymphoma with digital clubbing in a differential diagnosis.

## Author contributions

**Conceptualization:** Xiaoyan Gai, Wei Yang.

**Data curation:** Xiaoyan Gai, Wei Yang, Hong Zhu.

**Funding acquisition:** Xiaoyan Gai.

**Investigation:** Wei Yang, Hong Zhu.

**Methodology:** Wei Yang, Hong Zhu.

**Writing – original draft:** Xiaoyan Gai.

**Writing – review & editing:** Xiaoyan Gai, Yongchang Sun.
